# Mitochondrial fission protein Drp1 inhibition promotes cardiac mesodermal differentiation of human pluripotent stem cells

**DOI:** 10.1038/s41420-018-0042-9

**Published:** 2018-03-05

**Authors:** Ashfaqul Hoque, Priyadharshini Sivakumaran, Simon T. Bond, Naomi X. Y. Ling, Anne M. Kong, John W. Scott, Nadeeka Bandara, Damián Hernández, Guei-Sheung Liu, Raymond C. B. Wong, Michael T. Ryan, Derek J. Hausenloy, Bruce E. Kemp, Jonathan S. Oakhill, Brian G. Drew, Alice Pébay, Shiang Y. Lim

**Affiliations:** 1 St Vincent’s Institute of Medical Research, Fitzroy, VIC 3065 Australia; 2 Molecular Metabolism and Ageing Laboratory, Baker Heart and Diabetes Institute, Melbourne, VIC 3004 Australia; 3 School of Biomedical Sciences, Charles Sturt University, Wagga Wagga, NSW 2678 Australia; 4 Centre for Eye Research Australia, Royal Victorian Eye and Ear Hospital, East Melbourne, VIC 3065 Australia; 5 Departments of Medicine and Surgery, University of Melbourne, Melbourne, VIC 3065 Australia; 6 Menzies Institute for Medical Research, University of Tasmania, Hobart, Tasmania 7000 Australia; 7 Shenzhen Eye Hospital, Shenzhen, China; 8 Department of Biochemistry and Molecular Biology, Monash Biomedicine Discovery Institute, Monash University, Melbourne, VIC 3800 Australia; 9 Hatter Cardiovascular Institute, University College London, London, WC1E 6HX UK; 10 The National Institute of Health Research University College London Hospitals Biomedical Research Centre, London, UK; 11 Barts Heart Centre, St Bartholomew’s Hospital, London, UK; 12 Cardiovascular and Metabolic Disorders Program, Duke-National University of Singapore Medical School, Singapore, Singapore; 13 National Heart Research Institute Singapore, National Heart Centre, Singapore, Singapore; 14 Yong Loo Lin School of Medicine, National University Singapore, Singapore, Singapore; 15 Mary MacKillop Institute for Health Research, Australian Catholic University, Melbourne, VIC 3000 Australia

## Abstract

Human induced pluripotent stem cells (iPSCs) are a valuable tool for studying the cardiac developmental process in vitro, and cardiomyocytes derived from iPSCs are a putative cell source for personalized medicine. Changes in mitochondrial morphology have been shown to occur during cellular reprogramming and pluripotent stem cell differentiation. However, the relationships between mitochondrial dynamics and cardiac mesoderm commitment of iPSCs remain unclear. Here we demonstrate that changes in mitochondrial morphology from a small granular fragmented phenotype in pluripotent stem cells to a filamentous reticular elongated network in differentiated cardiomyocytes are required for cardiac mesodermal differentiation. Genetic and pharmacological inhibition of the mitochondrial fission protein, Drp1, by either small interfering RNA or Mdivi-1, respectively, increased cardiac mesoderm gene expression in iPSCs. Treatment of iPSCs with Mdivi-1 during embryoid body formation significantly increased the percentage of beating embryoid bodies and expression of cardiac-specific genes. Furthermore, Drp1 gene silencing was accompanied by increased mitochondrial respiration and decreased aerobic glycolysis. Our findings demonstrate that shifting the balance of mitochondrial morphology toward fusion by inhibition of Drp1 promoted cardiac differentiation of human iPSCs with a metabolic shift from glycolysis towards oxidative phosphorylation. These findings suggest that Drp1 may represent a new molecular target for future development of strategies to promote the differentiation of human iPSCs into cardiac lineages for patient-specific cardiac regenerative medicine.

## Introduction

Induced pluripotent stem cells (iPSCs) represent a major advance for potential autologous cell therapies. Cardiomyocytes derived from iPSCs can be used for cell-based therapy to treat heart disease, drug screening and cardiac disease modelling. Several agents have been shown to promote differentiation of pluripotent stem cells toward cardiomyogenic lineages, mainly through manipulation of the transforming growth factor-β (TGF-β), Nodal, Wnt, Notch and fibroblast growth factor signalling pathways^1,2^. However, the biological mechanisms governing commitment of iPSCs to cardiomyocyte lineage are still poorly understood. Thus, further understanding the cellular and molecular events guiding the cardiac differentiation of human iPSCs are crucial for developing more robust and reproducible cardiac differentiation protocols, as well as improving our understanding of key regulatory machineries of human heart development.

Mitochondria are dynamic organelles which continuously alter their morphology by undergoing fusion and fission processes to generate elongated and fragmented mitochondria, respectively. These two opposing processes are essential to maintain mitochondrial integrity and homoeostasis, and are regulated by a group of evolutionarily conserved mitochondrial fusion and fission proteins^3^. Studies have reported that mice lacking the mitochondrial fission (Drp1 and Mff) or fusion (Mfn1, Mfn2 and Opa1) proteins all exhibit developmental cardiac defects and increased susceptibility to cardiac injury^4,5^, underscoring the importance of mitochondrial dynamics in cardiac development and disease. Studies using iPSCs have reported a critical role of mitochondrial fusion and fission proteins in pluripotency induction^6,7^. In mouse embryonic fibroblasts, Mfn1/2 ablation facilitated somatic cell reprogramming to pluripotent stem cells^6^, whereas pharmacological inhibition of mitochondrial fission protein Drp1 limited cell fate conversion toward pluripotency^7^. Furthermore, mitochondrial fusion has been demonstrated to be essential for cardiac differentiation of murine embryonic stem cells, with embryonic stem cells deficient in mitochondrial fusion proteins Mfn2 and Opa1 having impaired ability to differentiate into cardiomyocytes^8^, indicating that mitochondrial morphology is an important regulator of cell fate.

In light of these findings, we hypothesized that inhibiting the dynamin-related GTPase Drp1, a key regulator of mitochondrial fission, can promote cardiac differentiation of iPSCs. To test this hypothesis, we employed siRNA-mediated gene silencing approach to knockdown Drp1 expression in human iPSCs. For timely manipulation of mitochondrial morphology, we employed Mdivi-1, a cell-permeable quinazolinone derivative small molecule, to reversibly inhibit Drp1, in both two-dimensional and three-dimensional cultures of iPSCs. For mechanistic insights, we studied the kinase profile of Mdivi-1 and changes in mitochondrial bioenergetics following inhibition of Drp1.

## Results

### Changes in mitochondrial morphology during cardiac differentiation

**Fig. 1:**
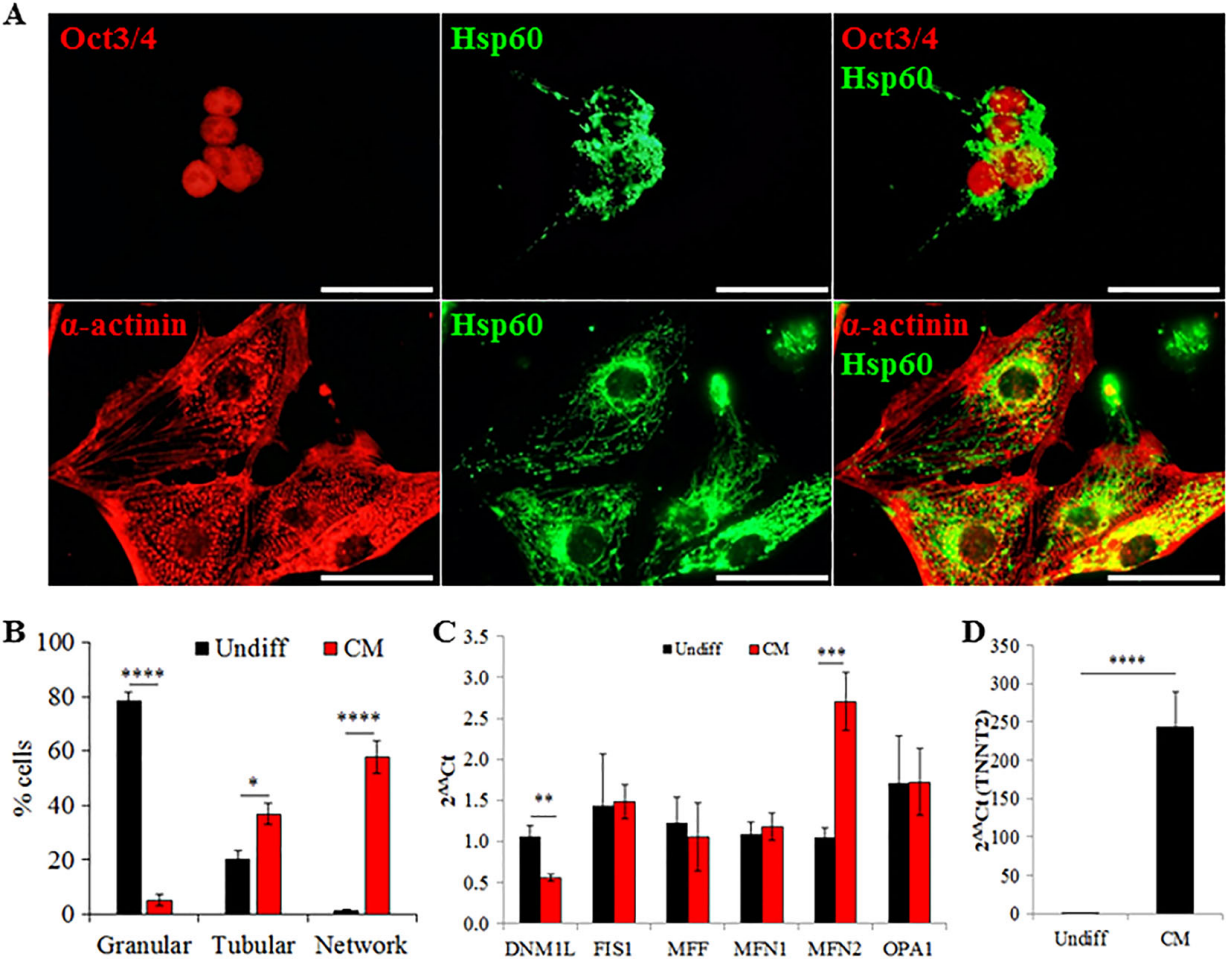
Mitochondrial morphology of human iPSCs and derived cardiomyocytes in iPS-Foreskin-2 cell line. **a** Morphology of mitochondria in undifferentiated iPSCs (Oct3/4-positive cells) and the derived cardiomyocytes (α-actinin-positive cells). Scale bars = 50 μm. **b** Percentage of cells with different mitochondrial morphologies (*n* = 4 independent experiment). **c, d** mRNA expression of mitochondrial fusion and fission genes (**c**), as well as cardiac-specific TNNT2 gene (**d**) in undifferentiated iPSCs (Undiff) and derived cardiomyocytes (CM) (*n* = 8 independent experiments). Data are shown as mean ± SEM. *P < 0.05, **P < 0.01, ***P < 0.001, ****P < 0.0001 by unpaired Student’s *t*-test

To investigate the regulation of mitochondrial morphology during cardiac differentiation of human iPSCs, the morphology of mitochondria and expression of genes related to mitochondrial morphology were compared between undifferentiated iPSCs and derived cardiomyocytes. In the pluripotent state, iPSCs (iPS-Foreskin-2 cell line) have fragmented and punctate mitochondria with perinuclear localization. As iPSCs differentiated into cardiomyocytes, the granular mitochondria became more tubular and formed net-like reticular networks throughout the cytoplasm (Fig. 1a, b), consistent with previous findings in embryonic stem cells showing substantial structural changes and distribution of mitochondria during differentiation^8^. These changes in morphology were accompanied by a significantly lower mRNA expression of the mitochondrial fission protein, Drp1 (*DNM1L*), and a higher mRNA expression of *MFN2* (a mitochondrial fusion protein) in the derived cardiomyocytes (Fig. 1c). The lower mRNA expression of Drp1 in cardiomyocytes compared to undifferentiated iPSCs was also observed in another iPSC line, CERA007c6. However, in CERA007c6 iPSCs, the mRNA expression of MFN2 was similar between undifferentiated iPSCs and the derived cardiomyocytes while the mRNA expression of MFN1 was significantly lower in the derived cardiomyocytes (Supplemental Figure S1A). Successful induction of cardiomyocytes from iPSCs was confirmed by spontaneous contraction and a significant up-regulation in expression level of cardiac troponin T, *TNNT2* (Fig. 1d; Supplemental Figure S1B). These observations prompted us to investigate the role of the mitochondrial fission protein Drp1 in regulating cardiac mesoderm differentiation of iPSCs.

### Drp1 gene silencing commits iPSCs toward cardiac mesoderm lineage

**Fig. 2:**
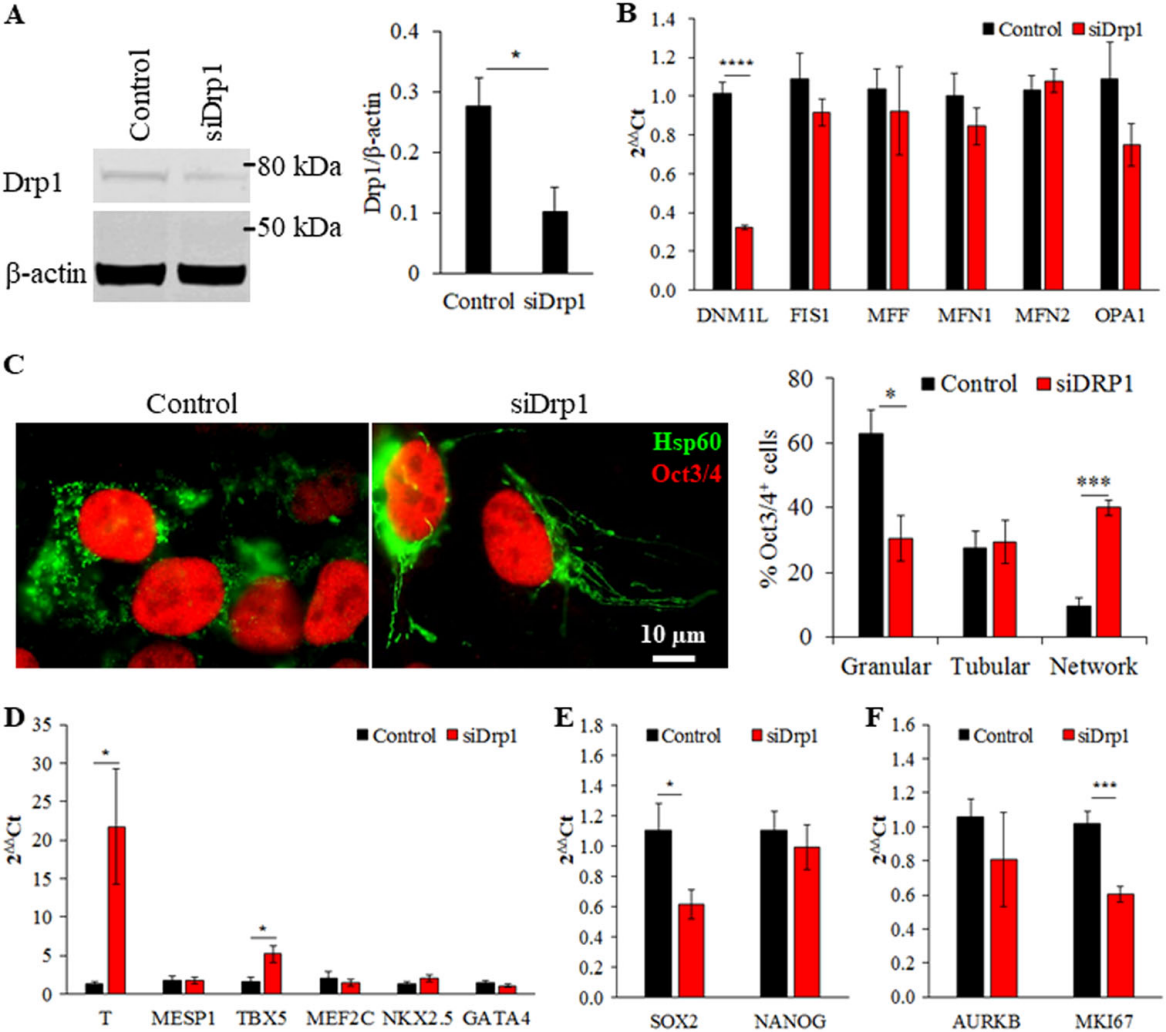
Drp1 knockdown in human iPSCs induces cardiac mesoderm differentiation. **a** Western blotting analysis of Drp1 expression (*n* = 4 independent experiments) and **b** RT-qPCR analysis of mitochondrial fusion and fission gene expression (*n* = 7–11 independent experiments) in iPS-Foreskin-2 cells treated with scrambled (control) or DRP1 siRNA. **c** Morphology of mitochondria and percentage of Oct3/4-positive cells with different mitochondrial morphologies (*n* = 4 independent experiment). **d–f** mRNA expression of cardiac mesoderm transcription factors (**d**), pluripotency genes (**e**) and cell proliferation genes (**f**) in iPS-Foreskin-2 cells treated with scrambled (control) or Drp1 siRNA (*n* = 8–11 independent experiments). Data are shown as mean ± SEM. *P < 0.05, ***P < 0.001, ****P < 0.0001 by unpaired Student’s *t*-test

To evaluate the functional role of Drp1 in iPSCs, undifferentiated iPSCs cultured in the TeSR-E8 pluripotency-maintaining medium were transiently transfected with Drp1 siRNA, which effectively reduced Drp1 protein and gene expression without affecting the expression of *FIS1*, *MFF, MFN1, MFN2* and *OPA1* (Fig. 2a, b, Supplemental Figure S2). Knockdown of Drp1 in iPSCs (iPS-Foreskin-2 cell line) for 2 days significantly increased the proportion of OCT3/4-positive cells with elongated mitochondria (Fig. 2c). This shift in mitochondrial morphology was accompanied by significant up-regulation in expression of early mesodermal gene brachyury (*T*) and cardiac progenitor gene *TBX5* (Fig. 2d), while the mRNA expression of ectodermal (*PAX6* and *TUBB3*) and endodermal (*AFP* and *CDH1*) markers was not significantly affected by Drp1 knockdown (Supplementary Figure S3). The mRNA expression of pluripotency factor *SOX2*, but not *NANOG*, was significantly down-regulated in Drp1-knockdown iPSCs (Fig. 2e). Analysis of mRNA expression levels of cell cycle markers showed reduced expression of *MIK67* but not *AURKB*, indicating reduced cell proliferation without affecting cytokinesis in Drp1-knockdown iPSCs (Fig. 2f). Similar results were obtained with CERA007c6 iPSC line, although increased expression of cardiac transcription factor *NKX2.5* and reduced expression of cell cycle regulatory gene *AURKB* were also detected in Drp1-knockdown CERA007c6 iPSCs (Supplemental Figure S2). The effect of Drp1 inhibition on iPSCs was further confirmed with Mdivi-1, a small molecule that prevents the GTPase activity of Drp1 and its polymerization at the outer mitochondrial membrane^9^. Two days treatment with Mdivi-1 significantly increased the proportion of OCT3/4-positive cells with tubular mitochondria and expression of *MFN2*, while the expression of other mitochondrial fission (*DNM1L*, *FIS1* and *MFF*) and fusion (*MFN1* and *OPA1*) genes were not affected (Supplemental Figure S4). Mdivi-1-treated cells also have higher expression of early mesodermal gene brachyury (*T*) and lower expression of cell cycle marker *MIK67* compared to the vehicle control group (Supplemental Figure S4). These data indicate that both genetic knockdown and pharmacological inhibition of Drp1 initiates cardiac mesoderm differentiation from human iPSCs.

### Pharmacological inhibition of Drp1 with Mdivi-1 promotes cardiac differentiation

**Fig. 3:**
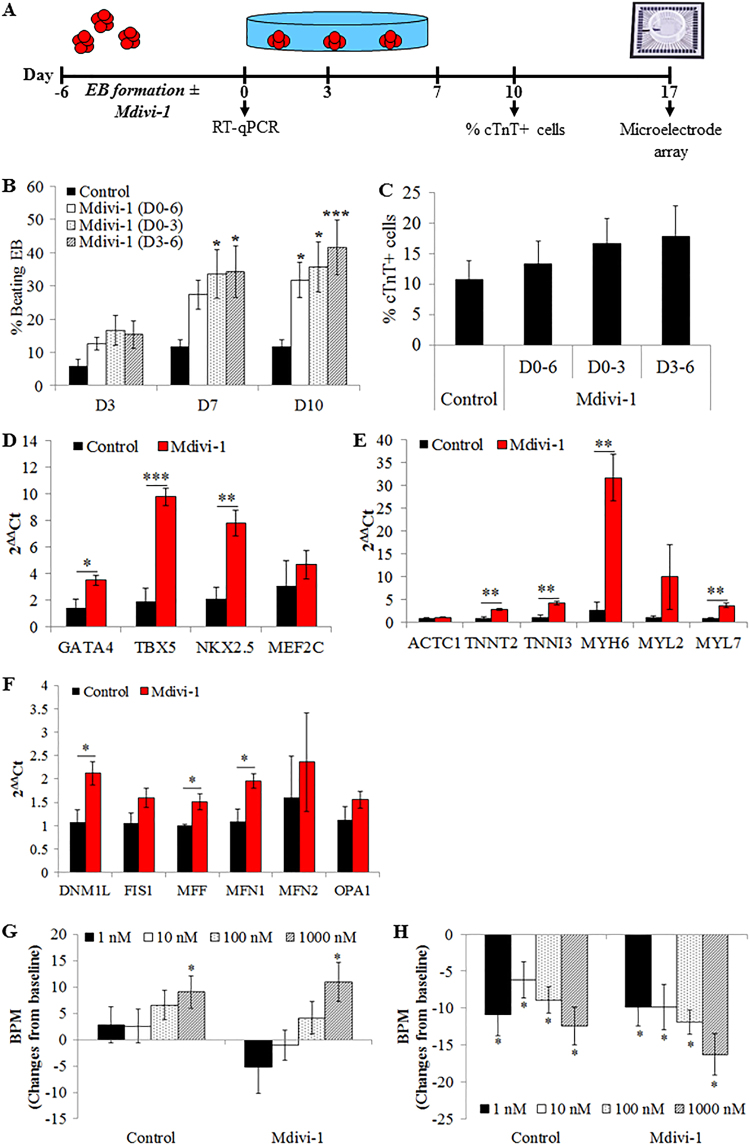
Mdivi-1 promotes cardiac differentiation of human iPSCs. **a** Schematic of the embryoid body-based cardiac differentiation protocol. **b** Effect of Mdivi-1 on the percentage of beating EBs (*n* = 7–8 independent experiments). **c** Percentage of cardiac troponin T-positive cells in each beating EB at day 10 post-plating (*n* = 8–10 independent experiments). **d–f** mRNA expression of cardiac mesoderm transcription factors (**d**), cardiac muscle proteins (**e**) and mitochondrial fusion and fission protein (**f**) (*n* = 4 independent experiments) in iPS-Foreskin-2 cells treated with DMSO (control) or 5 µM Mdivi-1 for 6 days during embryoid body formation. Changes in the beating rate of cardiomyocytes derived from control or Mdivi-1 groups treated with isoproterenol hydrochloride (isoprenaline, 1–1000 nM) (**g**) or carbamylcholine (carbachol, 1–1000 nM) (**h**) (*n* = 10 independent experiments). Data are expressed as mean ± SEM. *P < 0.05 and ***P < 0.001 vs. control (**a**–**f**) or baseline (**g**,** h**) by one-way ANOVA with the Bonferroni post hoc test

To further evaluate the role of Drp1 in cardiac differentiation of iPSCs, an EB-based spontaneous differentiation method was employed to rule out the potential confounding influence of cardiogenic factors needed for directed cardiac differentiation of iPSC in monolayer format. Limited by the poor transfection efficiency of three-dimensional structure of EBs with siRNA, Mdivi-1 was used to inhibit Drp1 during EB formation (Fig. 3a). Treatment of iPSCs (iPS-Foreskin-2 cell line) with 5 µM of Mdivi-1 during EB formation resulted in a 3- to 4-folds increase in the percentage of beating EBs at day 10 post-plating (Fig. 3b). Treatment duration did not significantly affect the pro-cardiogenic effect of Mdivi-1 since treatment during the first 3 days or the last 3 days of EB formation resulted in a similar increased in the percentage of beating EBs as 6 days treatment with Mdivi-1 (Fig. 3b). A similar increase in the percentage of beating EBs was also observed in CERA007c6 iPSC line albeit at a lower rate (percentage of beating EBs at day 10 post-plating = 2.8 ± 1.8% in control vs. 12.8 ± 3.7% in Mdivi-1 D0-6, *P* < 0.05, *n* = 5). Despite the increase in the percentage of beating EBs, the percentage of cardiac troponin T-positive cardiomyocytes within each beating EB at day 10 post-plating was similar among all treatment groups (Fig. 3c).

Quantitative PCR analysis confirmed directed cardiac differentiation of iPSCs (iPS-Foreskin-2 cell line) by Mdivi-1 treatment (for 6 days during EB formation), where expression of genes encoding cardiac transcription factor *GATA4*, *TBX5*and *NKX2.5* (Fig. 3d), as well as cardiac structural and contractile proteins *TNNT2*, *TNNI3*, *MYH6* and *MYL7* (Fig. 3e) were significantly up-regulated in 6-day-old EBs. In parallel, transcripts for mitochondrial fission proteins *DNM1L* and *MFF*, and mitochondrial fusion protein *MFN1* were significantly up-regulated in Mdivi-1-treated EBs compared to control group (Fig. 3f).

Beating EBs from both control and Mdivi-1-treated groups were capable of cycling calcium as determined by a fluorescent calcium indicator Fluo-4 AM. Extracellular field potentials measured by MEA showed an average contraction rate of 77 ± 3 bpm in the control group and 79 ± 4 bpm in the Mdivi-1-treated group. Isoproterenol (Fig. 3g) and carbamylcholine (Fig. 3h) produced a concentration-dependent positive and negative chronotropic responses, respectively, in both control and Mdivi-1-treated beating EBs. These results suggest that Mdivi-1 treatment did not affect the electrophysiological properties of cardiomyocytes derived from iPSCs.

### Specificity of Mdivi-1 towards Drp1

**Fig. 4:**
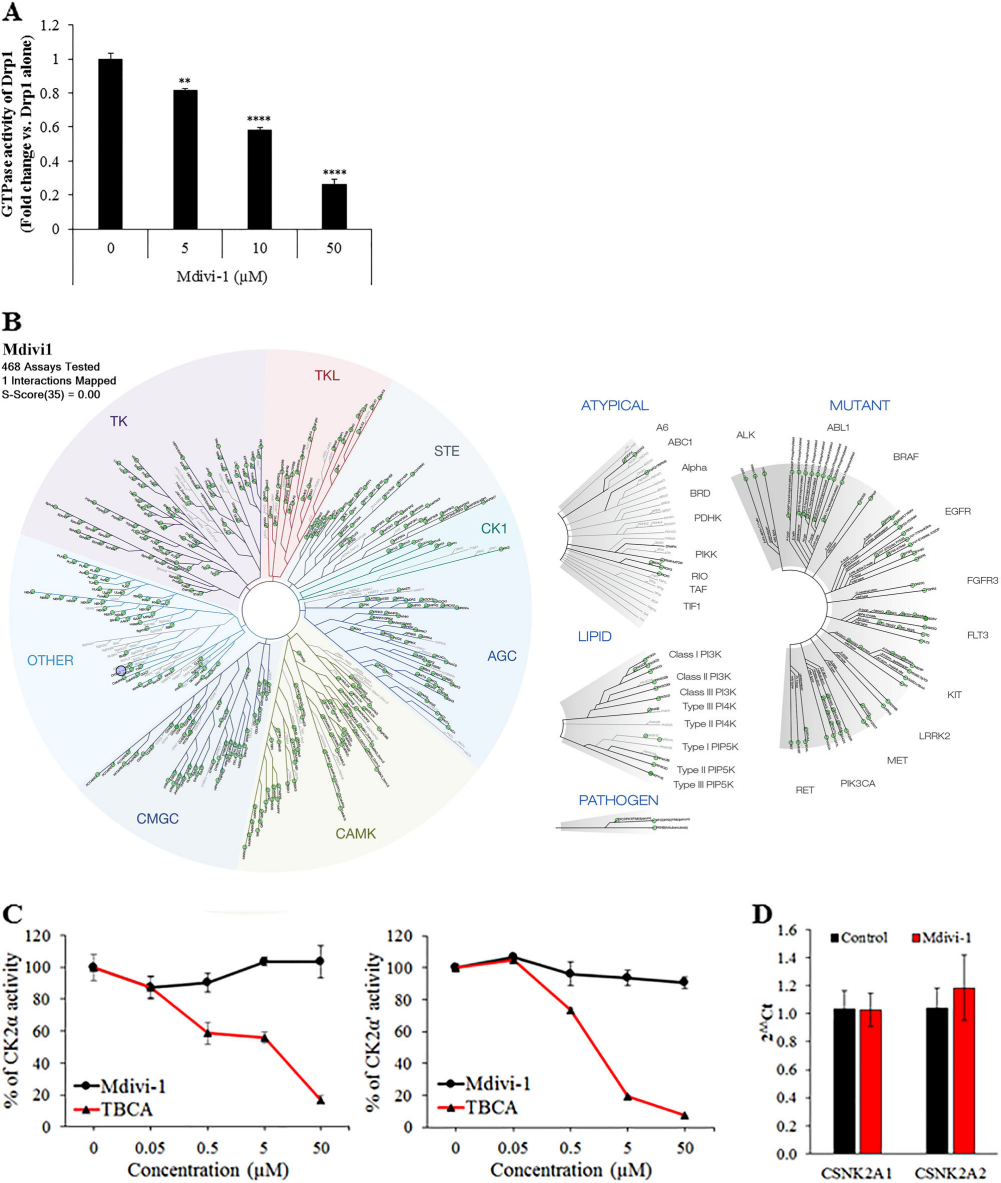
Effect of Mdivi-1 on Drp1 GTPase activity and other protein kinases. **a** GTPase activity of human recombinant Drp1 protein in the presence and absence of Mdivi-1. **b** Kinase interaction map of Mdivi-1 (10 µM) with 468 human protein kinases. The size of circle represents the relative binding score for each kinase. Mdivi-1 has one potential candidate kinase target, protein kinase CK2α′, with a binding score of 35% relative to the DMSO control. **c** Activities of protein kinase CK2α and CK2α′ in the presence or absence of Mdivi-1 or tetrabromocinnamic acid (TBCA) (*n* = 3). **d** mRNA expression of protein kinase CK2α and CK2α′ in iPS-Foreskin-2 cells treated with DMSO (control) or 5 µM Mdivi-1 (*n* = 9 independent experiments). Data are expressed as mean ± SEM. **P < 0.01, ***P < 0.001, ****P < 0.0001 by one-way ANOVA with the Bonferroni post hoc test

Mdivi-1 is a quinazolinone derivative that inhibits Drp1 assembly on the outer mitochondrial membrane by preventing the polymerization of higher order structures^9^. However, a recent study by Bordt et al.^10^ has shown that Mdivi-1 can inhibit respiratory complex I and this has challenged the specificity of Mdivi-1 as a selective inhibitor of Drp1. Using human recombinant Drp1 proteins, we showed that Mdivi-1 inhibits GTPase activity of Drp1 in a dose-dependent manner (Fig. 4a). To evaluate whether Mdivi-1 might also inhibit GTPase activity of Drp1 indirectly through interaction of other protein kinases, a high-throughput ATP-independent kinase assay was performed. Kinase screening against a panel of 468 human kinases with KINOMEscan assay showed a potential weak thermodynamic interaction between Mdivi-1 and protein kinase CK2α’ (*CSNK2A2*) (Fig. 4b, Supplementary Table S1) with a percent of control of 35%. To further assess this binding affinity of Mdivi-1 toward CK2 protein kinase, a cell-free in vitro CK2 kinase activity assay was performed using tetrabromocinnamic acid as a positive control. While tetrabromocinnamic acid exhibited a dose-dependent inhibition of CK2α and CK2α′ activities, Mdivi-1 did not significantly modulate CK2α and CK2α′ activities (Fig. 4c). In iPSCs, treatment with Mdivi-1 also did not affect the transcripts for CK2α (*CSNK2A1*) and CK2α′ (*CSNK2A2*) (Fig. 4d).

Despite in vitro insensitivity of CK2α activity to Mdivi-1, CK2α mRNA levels were significantly lower in differentiated cardiomyocytes compared to undifferentiated iPSCs (Supplemental Figure S5A). We considered that the pro-cardiogenic effect of Drp1 inhibition might involve protein kinase CK2. Knockdown of Drp1 in iPSCs did not change the mRNA and protein expression levels of CK2α and CK2α′ (Supplemental Figure S5B-C). Vice versa, knockdown of CK2α and CK2α′ did not significantly affect the mRNA expression of mitochondrial fusion and fission proteins or cardiac mesoderm-related transcription factors (Supplemental Figure S5D-F). Altogether, these data demonstrate that CK2 kinase is not involved in the pro-cardiogenic effect of Drp1 inhibition.

### Drp1 gene silencing induces metabolic switch

**Fig. 5:**
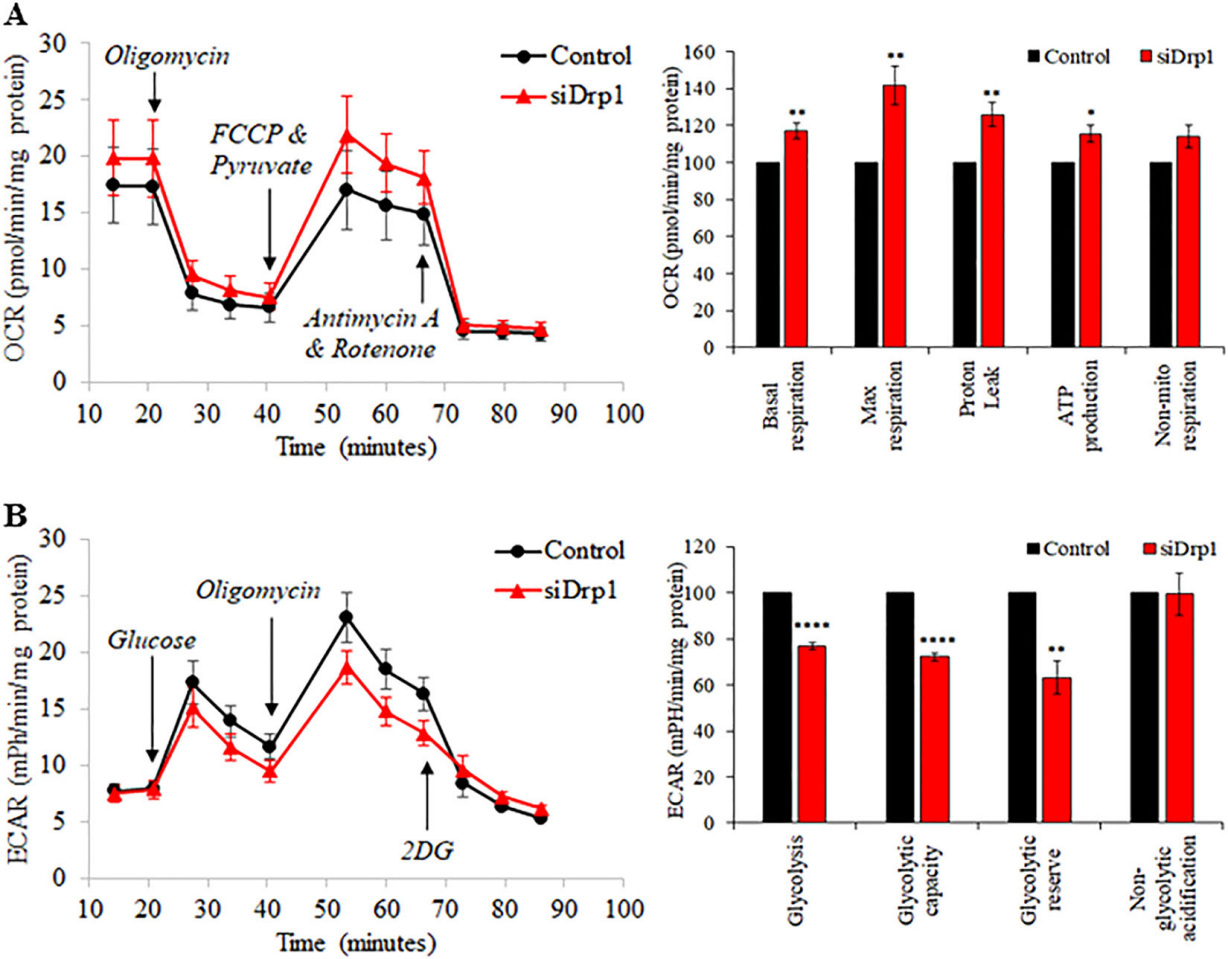
Drp1 knockdown in human iPSCs induces metabolic switch from glycolysis to oxidative phosphorylation. **a** Oxygen consumption rate (OCR) and **b** extracellular acidification rate (ECAR) of iPS-Foreskin-2 cells treated with scrambled (control) or Drp1 siRNA (*n* = 4 independent experiments). Data are shown as mean ± SEM. *P < 0.05, **P < 0.01, ****P < 0.0001 by unpaired Student’s *t*-test

Pluripotent stem cell metabolism is predominantly glycolytic which is in contrast to oxidative-based metabolism of cardiomyocytes^11^. To determine whether a metabolic switch from glycolysis to oxidative phosphorylation may underlie the pro-cardiogenic effect of Drp1 inhibition, the metabolic phenotypes of iPSCs were analysed using a Seahorse extracellular flux analyser with oxygen consumption rates (OCRs) and extracellular acidification rates (ECARs) as readouts for oxidative phosphorylation and glycolysis, respectively. Human iPSCs (iPS-Foreskin-2 cell line) showed oligomycin-sensitive oxygen consumption, and knockdown of Drp1 with siRNA significantly increased basal respiration (basal OCRs), maximal respiration, proton leak and ATP production, indicating increased oxidative phosphorylation (Fig. 5a). On the contrary, Drp1 gene silencing significantly reduced glycolysis, glycolytic capacity and glycolytic reserve in iPSCs (Fig. 5b). Interestingly, these changes in metabolic profiles were not recapitulated with Mdivi-1 treatment. Treatment with Mdivi-1 at a regimen (5 µM for 2 days) that promoted mitochondrial tubulation and cardiac mesodermal gene expression (Supplemental Figure S4) significantly reduced basal respiration, maximal respiration and ATP production in iPSCs, without significantly affecting glycolysis as determined by the ECAR (Supplemental Figure S6B). Neither Drp1 gene silencing nor Mdivi-1 treatment significantly affected the mRNA expression of peroxisome proliferator-activated receptor gamma coactivator 1α (*PPARGC1A*) and mitochondrial transcriptional factor-A (*TFAM*), two major regulators of mitochondrial biogenesis (Supplemental Figure S7). Overall these results suggest that Drp1 gene silencing induces a bioenergetics switch from glycolysis to oxidative metabolism.

## Discussion

Mitochondria are metabolic organelles that play an important role in various physiological functions including cell metabolism, proliferation, apoptosis and stem cell differentiation. The shape of mitochondria changes during cell fate conversion, from somatic cells with fused mitochondria to pluripotent stem cells with fragmented mitochondria and again in reverse as they differentiate into somatic cells^6,7,12^. This phenomenon highlights the importance of mitochondrial morphology in cell reprograming and stem cell differentiation. In this study, we uncovered a regulatory role of mitochondrial fission protein Drp1 in early cardiac mesodermal differentiation of human pluripotent stem cells, where inhibition of Drp1 promotes cardiac differentiation of iPSCs and this process was accompanied by a shift in mitochondrial dynamic equilibrium toward fusion and changes in mitochondrial bioenergetics from glycolysis to oxidative phosphorylation for energy production.

Drp1-mediated mitochondrial fission is implicated in maintenance of cell pluripotency and pluripotent cell reprogramming^13–15^. Inhibition of mitochondrial fission with either small interference RNA targeting Drp1, overexpression of a dominant-negative Drp1 or treatment with the Drp1 inhibitor Mdivi-1 diminishes the reprogramming efficiency of somatic cells into pluripotent stem cells^7,14^. As embryonic stem cells exit pluripotency and differentiate, mitochondria change their fragmented morphology and perinuclear localization to an elongated morphology that extends throughout the cytoplasm^12,16^. Similar morphological changes that we also observed in human iPSCs as they differentiate into cardiomyocytes. A previous study has implicated the importance of mitochondrial fusion proteins for cardiac development of murine cardiomyocytes by demonstrating that knockdown of pro-fusion proteins, Mfn2 and Opa1, impairs the ability of murine embryonic stem cells to undergo cardiac differentiation^8^. While they reported increase in Mfn2 and Opa1 expression as embryonic stem cells differentiate into cardiomyocytes, we detected a reduced expression of Drp1 in cardiomyocytes differentiated from human iPSCs. We further demonstrated that shifting the mitochondrial morphology toward fusion by silencing the expression of Drp1, or by inhibiting the activity of Drp1 with Mdivi-1, promotes cardiac mesodermal differentiation of human iPSCs. In addition to pluripotent stem cells, Drp1-dependent mitochondrial fission has also been implicated in maintaining the stemness of human nasopharyngeal carcinoma^17^, mouse skin mesenchymal stem cells^18^ and myogenic precursor cells^19^. These findings highlight the important role of mitochondrial dynamic in stem cell fate determination.

Metabolism is closely linked to cell fate^20^. Pluripotent stem cells have a high glycolytic flux and a relatively low mitochondrial respiration even when cultured in an oxygen-rich environment^21–23^. Compared to oxidative metabolism, glycolysis generates more energy and produces less reactive oxygen species and thus less DNA damage, which are essential for maintaining genomic integrity and healthy cell proliferation of pluripotent stem cells^24^. During the process of reprograming to iPSCs, somatic cells undergo a transition from mitochondrial respiration to aerobic glycolysis for more efficient generation of ATP and nucleotides to cope with the highly proliferative state of pluripotent stem cells^25,26^. Although impairment of Complex I mitochondrial respiration does not significantly impact on the efficiency of iPSC reprograming^27^, stimulation of glycolytic flux by glucose supplementation increases the efficiency of induced pluripotent reprogramming^25^. On the other hand, mitochondrial metabolism switches from aerobic glycolysis to favour oxidative phosphorylation-based metabolism when pluripotent stem cells exit the pluripotent state and differentiate^28,29^. Furthermore, this metabolic shift has been shown to occur prior to the loss of pluripotency^24^, and inhibition of glycolysis with glycolytic inhibitors upstream of acetate such as 2-deoxy-d-glucose and 3-bromopyruvate results in spontaneous differentiation of pluripotent stem cells^29^. Interestingly, a metabolic switch away from glycolysis during pluripotent stem cell differentiation has been shown to be specific for mesoderm and endoderm lineages while early ectoderm differentiation requires maintenance of high glycolytic flux and is regulated by MYC transcription factors^30^. In human iPSCs, we showed a shift in metabolism from glycolysis toward oxidative phosphorylation when Drp1 is knocked down. This metabolic change was associated with increased expression of cardiac mesodermal markers and reduced expression of pluripotency factor Sox2, demonstrating the close relationship between mitochondrial morphology, energy metabolism and pluripotency. This is in agreement with previous studies that reported an increase in mitochondrial oxidative respiration and mitochondrial elongation when stem cells lose their pluripotency^16^, while induction of mitochondrial fission, either via activation of mitochondrial fission protein Drp1 or by depletion of mitochondrial fusion protein Mfn1/2, induces metabolic shift in dependency of aerobic glycolysis to oxidative phosphorylation and promotes reprograming of somatic cells into iPSCs^6,13^.

Mdivi-1 is a quinazolinone derivative and a selective cell-permeable allosteric inhibitor of Drp1. Mechanistically, Mdivi-1 inhibits GTPase activity of Drp1 and prevents their assembly on the outer mitochondrial membrane by preventing the polymerization of higher order structures^9,31^. Although Mdivi-1 has been shown to inhibit GTPase activity of Dnm1, the yeast homologue of the mammalian Drp1^9^, the GTPase inhibitory effect of Mdivi-1 on recombinant human Drp1 has been controversial^9,10^. Using a different source of recombinant human Drp1 (isoform 2) that has 76% of amino acid sequence matching endogenous Drp1, we observed a significant reduction of GTPase activity of the recombinant human Drp1 when pre-incubated with Mdivi-1 for at least 30 min. This suggests the equilibrium binding interaction between Mdivi-1 and Drp1 required time to establish in a cell-free environment. Interestingly, while Mdivi-1 recapitulated the effect of Drp1 silencing in promoting mitochondrial fusion and cardiac mesodermal differentiation in human iPSCs, Mdivi-1 did not induce a metabolic shift as observed with Drp1 knockdown. Instead, treatment with Mdivi-1 at 5 µM resulted in significant reduction in mitochondrial respiration. In fact, a recent study in mouse embryonic fibroblasts has demonstrated that Mdivi-1 at a much higher concentration of 50 µM can reversibly inhibit mitochondrial complex I-dependent oxygen consumption and reverse electron transfer-mediated reactive oxygen species production in a Drp1-independent manner^10^. However, the inhibitory effect of Mdivi-1 on mitochondrial respiration could be cell type specific given that Mdivi-1 does not significantly modify mitochondrial oxygen consumption in human cardiac stem cells^32^. Although Mdivi-1 did not significantly interact with any of the 468 human protein kinases in our kinase profiling assay, this finding does not exclude the possibility that Mdivi-1 may interact with other protein classes such as other hydrolase enzymes, G-protein coupled receptors, ion channels, nuclear receptors, transcription factors and plasma proteins, which warrants further investigation.

In conclusion, manipulation of mitochondrial morphology by inhibiting Drp1 promotes cardiac mesodermal differentiation of human iPSCs, highlighting the regulatory importance of Drp1-mediated mitochondrial fission in maintaining stem cell pluripotency and controlling cell fate decisions.

## Methods

### Human iPSC culture and cardiac differentiation

The iPS-Foreskin-2 cell line was obtained from Prof J. Thompson (University of Wisconsin, USA)^33^. CERA007c6 iPSC line was generated using skin fibroblasts from a 36 years old healthy male by the episomal method as described previously^34^. Human iPSCs were maintained on a feeder layer of mitotically inactivated human foreskin fibroblasts (HFF:D551; ATCC, VA, USA) in Dulbecco's modified Eagle's medium (DMEM)/F-12 GlutaMAX medium supplemented with 20% knockout serum replacement, 0.1 mM 2-mercaptoethanol, 0.1 mM nonessential amino acids, 50 U/mL penicillin/streptomycin (all from Thermo Fisher Scientific, VIC, Australia) and 20 ng/mL recombinant human fibroblast growth factor-2 (Merck Millipore, CA, USA)^35^. For feeder-free culture, human iPSCs were maintained and propagated on vitronectin-coated plate in TeSR-E8 medium (Stem Cell Technologies, VA, Canada) according to the manufacturer’s protocol.

Spontaneous differentiation of iPSCs was induced through formation of embryoid bodies (EBs) as previously described^35^. Briefly, EBs were formed by mechanically dissecting undifferentiated iPSC colonies maintained on a feeder layer into approximately 0.2 mm^2^ pieces. Pieces were transferred onto low attachment plates and cultured in suspension in differentiation medium containing DMEM/F-12 GlutaMAX medium supplemented with 20% foetal bovine serum (Sigma-Aldrich, MO, USA), 0.1 mM 2-mercaptoethanol, 0.1 mM nonessential amino acids and 50 U/mL penicillin/streptomycin, where they aggregated to form EBs over 6 days. During EB formation, cells were treated with either 0.05% DMSO as vehicle control or 5 µM Mdivi-1 (Enzo Life Sciences, NY, USA). On day 6 (day 0 post-plating), EBs were transferred to tissue culture plates pre-coated with 0.1% gelatin (Sigma-Aldrich), and cultured in differentiation medium. The percentage of contractile EBs was measured as the number of EBs that showed spontaneous contraction divided by the total number of EBs plated.

For directed cardiac differentiation, iPSCs maintained on feeder-free culture were dissociated into single cells with TrypLE (Thermo Fisher Scientific) and seeded onto Matrigel (Corning, MA, USA) coated plate at a density of 1 × 10^5^ cells/cm^2^ in TeSR-E8 medium supplemented with 10 µM Y-27632 (Tocris Bioscience, Bristol, UK). After 2 days when the cells were 100% confluent, which is referred to as day 0, medium was replaced with RPMI 1640 basal medium containing B-27 without insulin supplement (Thermo Fisher Scientific), growth factor reduced Matrigel (1:60 dilution) and 10 µM CHIR99021 (Cayman Chemical, MI, USA). After 24 h, cells were treated with 5 ng/mL Activin A (Peprotech, NJ, USA) in RPMI 1640 basal medium containing B-27 without insulin supplement for 24 h. At day 2, the medium was changed to RPMI 1640 basal medium containing B-27 without insulin supplement and 5 µM IWP2 (Tocris Bioscience) for 72 h. From day 5 onward, cells were cultured in RPMI 1640 basal medium containing B-27 supplement (Thermo Fisher Scientific) and 200 µg/mL l-ascorbic acid 2-phosphate sesquimagnesium salt hydrate (Sigma-Aldrich).

### Microelectrode array recordings

The electrophysiological properties of the cardiomyocytes derived from iPS cells were evaluated using a microelectrode array (MEA) recording system (Multichannel Systems, Reutlingen, Germany). Beating cell clusters at day 10 post-plating were transferred onto MEA plates coated with 0.1% gelatin and 10 µg/mL fibronectin. Responsiveness to pharmacological agents was determined 4–6 days later at 37 °C in Krebs-Ringer buffer (composition in mM: 125 NaCl, 5 KCl, 1 Na_2_HPO_4_, 1 MgSO_4_, 20 HEPES, 5.5 glucose, 2 CaCl_2_; pH 7.4). The responsiveness of cells to isoproterenol hydrochloride (1–1000 nM, Sigma-Aldrich) and carbamylcholine (1–1000 nM, Sigma-Aldrich) was tested. Each cell cluster was treated with all drugs in random order and cells were allowed to recover to their baseline contraction in fresh Krebs-Ringer buffer in between drug treatments. Extracellular field potentials were recorded at baseline and 2 min after addition of drugs. Data were analysed offline with MC Rack version 4.3.5 software for beating rate, RR interval and extracellular field potential duration (FPD) as previously described^35,36^. FPD measurements were normalized (corrected FPD, cFPD) with the Bazzet correction formula: cFPD = FPD/√(RR interval).

### Small interfering RNA knockdown assay

iPSCs maintained on feeder-free culture were trypsinized and seeded on Matrigel-coated surface at a density of 5 × 10^4^ cells/cm^2^ in TeSR-E8 medium supplemented with 10 µM Y-27632. After overnight incubation, cells were transfected with corresponding siRNA using the Lipofectamine RNAiMAX transfection agent (Thermo Fisher Scientific) for 2 days in TeSR-E8 medium. For knockdown experiments, Drp1 (*DNM1L*) was silenced with 50 pmol of siRNA duplex (sc-43732; Santa Cruz Biotechnology, TX, USA). CK2α (*CSNK2A1*) and CK2α′ (*CSNK2A2*) were silenced with 50 pmol of ON-TARGETplus SMARTpool siRNA system (Thermo Fisher Scientific) which contains a pool of four different siRNAs.

### Immunocytochemistry

Cells were fixed in 4% paraformaldehyde and permeabilized with 0.2% triton X-100. After blocking with serum-free blocking solution (Thermo Fisher Scientific) for 10 min, cells were incubated with primary antibodies against Oct3/4 (20 µg/mL, mouse monoclonal IgG; Santa Cruz Biotechnology, TX, USA), cardiac troponin T (cTnT, 2 µg/mL, mouse monoclonal IgG; Abcam, MA, USA), α-actinin (25 µg/mL, mouse monoclonal IgG, A7811; Sigma-Aldrich), or Hsp60 (1.58 µg/mL, rabbit polyclonal IgG, Abcam) at 4 °C overnight followed by Alexa Fluor 488 goat anti-mouse IgG, Alexa Fluor 488 goat anti-rabbit IgG or Alexa Fluor 594 goat anti-mouse IgG (10 µg/mL; Thermo Fisher Scientific) for 60 min at room temperature. Cells were counterstained with 1 µg/mL DAPI (Thermo Fisher Scientific) for nuclear staining and mounted with fluorescence mounting agent (DAKO, Victoria, Australia). Images were acquired with a BX-61 Olympus fluorescence microscope (Tokyo, Japan).

For quantitative assessment of cardiomyocytes differentiation, spontaneously beating colonies at 10 days post-plating were trypsinized into single-cell suspension with 0.25% trypsin-EDTA and spun onto coated glass slides (4 min at 900 rpm; Shandon Cytospin 4; Thermo Fisher Scientific) and immunostained with cardiac troponin T followed by Alexa Fluor 488 goat anti-mouse IgG. Cells were counterstained with DAPI and mounted with fluorescence mounting agent. Images were taken with a BX-61 Olympus fluorescence microscope and at least 500 cells were counted.

### Real-time quantitative PCR (RT-qPCR)

RNA was extracted from cells using TriReagent (Thermo Fisher Scientific) followed by RNA precipitation with chloroform and isopropanol (Sigma-Aldrich). cDNA was synthesized using the high-capacity cDNA reverse transcription kit (Applied Biosystems, CA, USA). qPCR was carried out using TaqMan Universal master mix, the 7900HT Fast Real-Time PCR system and TaqMan gene expression assays (Applied Biosystems) for *GAPDH* (Hs03929097_g1), *T* (Hs00610080_m1), *MESP1* (Hs00251489_m1), *TBX5* (Hs00361155_m1), *GATA4* (Hs00171403_m1), *NKX2.5* (Hs00231763_m1), *MEF2C* (Hs00231149_m1), *ACTC1* (Hs01109515_m1), *TNNT2* (Hs01109515_m1), *TNNI3* (Hs00165957_m1), *MYH6* (Hs01101425_m1), *MYH7* (Hs00165276_m1), *MYL2* (Hs00166405_m1), *MYL7* (Hs01085598_g1), *AURKB* (Hs00945855_g1), *MIK67* (Hs01032443_m1), *NANOG* (Hs04260366_g1), *SOX2* (Hs01053049_s1), *DNM1L* (Hs00247147_m1), *FIS1* (Hs00211420_m1), *MFF* (Hs00697394_g1), *MFN1* (Hs00966851_m1), *MFN2* (Hs00208382_m1), *OPA1* (Hs01047013_m1),*CSNK2A1* (Hs00751002_s1), *CSNK2A2* (Hs00176505_m1), *TFAM* (Hs00273372_s1), *PPARGC1A* (Hs00173304_m1), *PAX6* (Hs01088112_m1), *TUBB3* (Hs00801390_s1), *AFP* (Hs01040598_m1) and *CDH1* (Hs01023894_m1). All readings were performed in duplicate. The relative quantitation was calculated by applying the comparative CT method (2^−ΔΔCt^) whereby the mRNA expression levels were normalized against the level of the housekeeping human gene *GAPDH* (^Δ^Ct) with the level of candidate genes in control samples used as the reference (^ΔΔ^Ct).

### Western blotting

Proteins from iPSCs were extracted with RIPA lysis buffer (Sigma-Aldrich) supplemented with protease inhibitor cocktail (Sigma-Aldrich). Proteins were denatured in NuPAGE LDS sample buffer (Thermo Fisher Scientific) and separated by SDS-PAGE using NuPAGE 12% Bis-Tris protein gels (Thermo Fisher Scientific) in NuPAGE MES SDS running buffer (Thermo Fisher Scientific). Proteins were then transferred onto a polyvinylidene difluride membrane (Amersham Hybond; GE Healthcare Life Sciences, NSW, Australia) and blocked with Odyssey blocking buffer (LI-COR Biosciences, NE, USA) for 30 min at room temperature. Following successive washes in phosphate-buffered saline (PBS) containing 0.1% Tween-20 (PBS-T), membranes were incubated with the following primary antibodies diluted in PBS-T: mouse monoclonal Drp1 (1 µg/mL; Abcam), mouse monoclonal CKII-alpha (4 µg/mL; Abcam), rabbit polyclonal CKII-alpha prime (2 µg/mL; Abcam) or mouse monoclonal β-actin (1:1000 dilution; LI-COR Biosciences) at 4 °C overnight. After three washes in PBS containing 0.1% Tween-20, membranes were incubated with appropriate fluorescently labelled secondary antibodies, either Alexa fluor-680 donkey anti-rabbit (0.1 µg/mL; Thermo Fisher Scientific) or IRDye^®^ 800CW goat anti-mouse (0.05 µg/mL; LI-COR Biosciences) for 1 h at room temperature. The membranes were scanned with Odyssey infrared imaging system (LI-COR Biosciences) and protein band intensity was determined by computerized densitometry using NIH Image J software and expressed as arbitrary units after normalized with β-actin.

### KINOMEscan

The active site-directed competition binding assays were performed using the KINOME*scan* kinase profiling platform (DiscoverX, CA, USA). KINOMEscan assay do not require ATP and thus report true thermodynamic interaction affinities^37^. The assays measured the interactions of Mdivi-1 (10 µM in 0.1% DMSO) with 468 kinases covering more than 80% of the human kinome and disease-relevant mutant variants. Kinase dendrogram was generated using TREE*spot* software (http://www.kinomescan.com). The readout from KINOMEscan assay is ‘percent of control’, where the control is 0.1% DMSO and 100% indicates no inhibition of the kinase by Mdivi-1 at 10 µM.

### Recombinant his6-Drp1 production and purification

For bacterial expression of recombinant human Drp1 (N-term his6) protein, cDNA for human Drp1 (Isoform 2, Uniprot ID: O00429-3) was cloned into pQE-30 vector and transformed in Rosetta (DE3) competent cells (Novagen, Merck Millipore). Transformed cells were propagated in a shaker incubator in 2 L Luria-Bertani broth, in the presence of 100 µg/mL ampicillin at 37 °C, 120 rpm to *A*_600_ = 2–2.5. Protein expression was induced by addition of 0.5 mM IPTG, after which cultures were incubated at 16 °C for 20–22 h. Cells were harvested by centrifugation at 3500 rpm, 20 min and the pellet re-suspended in lysis buffer containing 50 mM Tris.HCl (pH 7.3), 0.5 M NaCl, 50 mM imidazole, 5% glycerol, 2 mM β-mercaptoethanol, 0.1 mM LEUPEP, 0.1 mM AEBSF and 1 mM benzaminidium chloride. Cell were lysed using a pre-cooled EmulsiFlex-C5 homogenizer (Avestin, Ottawa, Canada). Cell lysates were clarified by centrifugation at 20,000 rpm for 30 min and loaded onto a 5 mL Nickel Chelating Sepharose Fast Flow column (GE Healthcare, Buckinghamshire, UK). The column was washed with 20 column volumes of lysis buffer without addition of protease inhibitors. Drp1 protein was eluted with 50 mM Tris.HCl (pH 7.6), 150 mM NaCl, 400 mM imidazole, 10% glycerol and 2 mM β-mercaptoethanol and exchanged into storage buffer (50 mM Tris.HCl (pH 7.6), 150 mM NaCl, 10% glycerol, 2 mM TCEP) using a PD-10 desalting column (GE Healthcare) and aliquots stored at −80 °C.

### Mammalian cell expression of HA-tagged CK2α and CK2α′

CK2α (Uniprot P68400, C-terminal HA-tagged, Addgene plasmids #27086) and CK2α′ (Uniprot P19784, N-terminal HA-tagged, Addgene plasmids #27087) proteins (gifts from David Litchfield) were expressed in COS7 cells which were cultured in DMEM medium supplemented with 10% foetal bovine serum and incubated at 5% CO_2_ at 37 °C. Cells were transfected with 2 μg plasmid DNA per 10 cm culture dish, using FuGENE HD (Promega, WI, USA) according to the manufacturer’s instruction. Cells were harvested 48 h post-transfection and lysed in ice cold 50 mM Tris-HCl (pH 7.4), 50 mM NaCl, 10% glycerol, 1 mM EDTA, 1 mM EGTA, 5 mM sodium pyrophosphate, 50 mM NaF, 1% Triton X-100 and cOmplete protease inhibitor cocktail (Roche, Basel, Switzerland). Lysates were clarified by centrifugation (14,000 rpm, 10 min, 4 °C). Supernatants containing expressed CK2 proteins were used for subsequent immunoprecipitation experiments or stored at −80 °C for further use.

### CK2 kinase activity assay

CK2 kinase activity was assayed using radiolabelled ^32^P-ATP and synthetic peptide substrate CKtide (RRRDDDSDDD-NH_2_). CK2α and CK2α′ proteins from prepared lysates was immobilized on anti-HA affinity gel (Sigma-Aldrich) and washed four times with wash buffer (50 mM HEPES (pH 7.4), 150 mM NaCl, 10% glycerol, 1 mM DTT and 0.1% Tween-20) prior to the kinase reaction. Activity assay was conducted in the presence of 200 µM CKtide peptide, 5 mM MgCl_2_, 200 µM [γ-^32^P] ATP for 10 min at 30 °C, in the presence or absence of 0.05–50 µM Mdivi-1 or tetrabromocinnamic acid (Calbiochem, MA, USA). Phosphotransferase activity was quenched by spotting 15 μL onto P81 phosphocellulose paper (Whatman; GE Healthcare, Little Chalfont, UK), followed by repeated washes in 1% (v/v) phosphoric acid. ^32^P transfer was quantified by liquid scintillation counting (PerkinElmer, MA, USA).

### GTPase activity assay

Recombinant human Drp1 was incubated with Mdivi-1 at 37 °C for 30 min and GTPase activity of Drp1 was determined using the GTPase assay kit (Novus Biologicals, CO, USA) according to the manufacturer’s instructions.

### Analysis of mitochondrial respiration and glycolysis

All extracellular flux analyses were performed using the Seahorse XFe96 Extracellular Flux Analyser (Agilent, Santa Clara, USA). Briefly, iPSCs were plated at 5 × 10^4^ cells/well in a 96-well Seahorse V3-PS plate pre-coated with growth factor reduced Matrigel (1:60 dilution). After overnight incubation and prior to assay, cells were washed with assay media (Seahorse XF base medium supplemented with 25 mM glucose, 1 mM glutamine and 1 mM sodium pyruvate for OCR assay, or Seahorse XF base medium supplemented with 1 mM glutamine for ECAR assay), and equilibrated in 175 μL of respective assay media per well at 37 °C with no CO_2_ for 30 min. The assay protocol consisted of repeated cycles of 3 min mix and 3 min measurement periods, with OCR and ECAR measured simultaneously. Basal energetics were established after four of these initial cycles, followed by performing a mitochondrial stress test which includes sequential injection of the following compounds and subsequent measurement of OCR after each: the ATP synthase inhibitor oligomycin (1 μM), the proton ionophore carbonyl cyanide-4-(trifluoromethoxy)phenylhydrazone (FCCP, 125 nM) together with sodium pyruvate (1 mM), and the mitochondrial complex III and Complex I inhibitors antimycin A/rotenone (1 µM). The glycolysis stress test includes sequential injection of the following compounds and subsequent measurement of ECAR after each: glucose (10 mM), oligomycin (1 μM), and 2-deoxy-glucose (2DG, 50 mM). All treatment conditions were analysed as 2–4 replicates for each independent experiment. At the completion of each assay, cells were lysed and protein concentration was determined using the Bradford dye-binding method (Bio-Rad, Sydney, Australia) according to the manufacturer’s instructions.

### Statistics

All values are expressed as mean ± standard error of the mean (SEM). Significance of the differences was evaluated using unpaired Student’s *t*-test or one-way ANOVA followed by Bonferroni multiple comparison post hoc analyses where appropriate. *P* < 0.05 was considered statistically significant.

## Electronic supplementary material

Supplementary table and figures
