# Supplementary material for: Mitochondrial fission protein Drp1 inhibition promotes cardiac mesodermal differentiation of human pluripotent stem cells
Source: Cell Death Discov. 2018 Mar 5;4:39. doi: 10.1038/s41420-018-0042-9 (PMC5841367; doi:10.1038/s41420-018-0042-9)
Supplement: Supplementary file 1 — Supplementary table and figures [file 41420_2018_42_MOESM1_ESM.pdf]

Supplementary Information

Mitochondrial fission protein Drp1 inhibition promotes cardiac mesodermal differentiation of human pluripotent stem cells

Ashfaqul Hoque, Priyadharshini Sivakumaran, Simon T. Bond, Naomi X.Y. Ling, Anne M. Kong, John Scott, Nadeeka Bandara, Damián Hernández, Guei-Sheung Liu, Raymond C.B. Wong, Michael T. Ryan, Derek J. Hausenloy, Bruce E. Kemp, Jonathan S. Oakhill, Brian G. Drew, Alice Pébay, Shiang Y. Lim

Supplemental Table S1. Kinase profiling of Mdivi-1 at 10 µM by KINOMEscan assay.

DiscoveRx Gene Symbol	Percent Control	DiscoveRx Gene Symbol	Percent Control
AAK1	95	BRAF	96
ABL1(E255K)-phosphorylated	71	BRAF(V600E)	72
ABL1(F317I)-nonphosphorylated	95	BRK	84
ABL1(F317I)-phosphorylated	100	BRSK1	94
ABL1(F317L)-nonphosphorylated	92	BRSK2	93
ABL1(F317L)-phosphorylated	92	BTK	88
ABL1(H396P)-nonphosphorylated	88	BUB1	100
ABL1(H396P)-phosphorylated	83	CAMK1	77
ABL1(M351T)-phosphorylated	95	CAMK1B	74
ABL1(Q252H)-nonphosphorylated	64	CAMK1D	85
ABL1(Q252H)-phosphorylated	100	CAMK1G	98
ABL1(T315I)-nonphosphorylated	96	CAMK2A	97
ABL1(T315I)-phosphorylated	83	CAMK2B	100
ABL1(Y253F)-phosphorylated	88	CAMK2D	100
ABL1-nonphosphorylated	73	CAMK2G	86
ABL1-phosphorylated	81	CAMK4	90
ABL2	92	CAMKK1	95
ACVR1	94	CAMKK2	76
ACVR1B	96	CASK	83
ACVR2A	100	CDC2L1	89
ACVR2B	100	CDC2L2	100
ACVRL1	83	CDC2L5	88
ADCK3	94	CDK11	100
ADCK4	100	CDK2	91
AKT1	89	CDK3	100
AKT2	100	CDK4	86
AKT3	100	CDK4-cyclinD1	94
ALK	91	CDK4-cyclinD3	100
ALK(C1156Y)	70	CDK5	100
ALK(L1196M)	79	CDK7	95
AMPK-alpha1	100	CDK8	96
AMPK-alpha2	87	CDK9	94
ANKK1	97	CDKL1	100
ARK5	99	CDKL2	100
ASK1	99	CDKL3	98
ASK2	84	CDKL5	89
AURKA	82	CHEK1	90
AURKB	86	CHEK2	89
AURKC	88	CIT	94
AXL	100	CLK1	95
BIKE	93	CLK2	100
BLK	95	CLK3	97
BMPR1A	97	CLK4	92
BMPR1B	86	CSF1R	95
BMPR2	84	CSF1R-autoinhibited	100
BMX	100	CSK	97

CSNK1A1	71	EPHB4	86
CSNK1A1L	88	EPHB6	90
CSNK1D	78	ERBB2	100
CSNK1E	92	ERBB3	89
CSNK1G1	100	ERBB4	90
CSNK1G2	88	ERK1	94
CSNK1G3	92	ERK2	100
CSNK2A1	52	ERK3	88
CSNK2A2	35	ERK4	95
CTK	100	ERK5	95
DAPK1	100	ERK8	96
DAPK2	95	ERN1	100
DAPK3	85	FAK	94
DCAMKL1	94	FER	95
DCAMKL2	100	FES	89
DCAMKL3	77	FGFR1	79
DDR1	90	FGFR2	84
DDR2	94	FGFR3	97
DLK	97	FGFR3(G697C)	87
DMPK	100	FGFR4	82
DMPK2	88	FGR	79
DRAK1	98	FLT1	85
DRAK2	95	FLT3	100
DYRK1A	71	FLT3(D835H)	97
DYRK1B	83	FLT3(D835V)	43
DYRK2	97	FLT3(D835Y)	78
EGFR	95	FLT3(ITD)	83
EGFR(E746-A750del)	80	FLT3(ITD,D835V)	67
EGFR(G719C)	67	FLT3(ITD,F691L)	65
EGFR(G719S)	52	FLT3(K663Q)	93
EGFR(L747-E749del, A750P)	94	FLT3(N841I)	78
EGFR(L747-S752del, P753S)	83	FLT3(R834Q)	87
EGFR(L747-T751del,Sins)	69	FLT3-autoinhibited	100
EGFR(L858R)	99	FLT4	100
EGFR(L858R,T790M)	100	FRK	86
EGFR(L861Q)	65	FYN	81
EGFR(S752-I759del)	40	GAK	83
EGFR(T790M)	85	GCN2(Kin.Dom.2,S808G)	100
EIF2AK1	100	GRK1	96
EPHA1	87	GRK2	97
EPHA2	91	GRK3	100
EPHA3	97	GRK4	96
EPHA4	100	GRK7	100
EPHA5	99	GSK3A	94
EPHA6	98	GSK3B	84
EPHA7	97	HASPIN	90
EPHA8	95	HCK	92
EPHB1	99	HIPK1	82
EPHB2	100	HIPK2	92
EPHB3	98	HIPK3	70

HIPK4	88	MAP4K4	100
HPK1	98	MAP4K5	100
HUNK	94	MAPKAPK2	91
ICK	96	MAPKAPK5	99
IGF1R	90	MARK1	95
IKK-alpha	94	MARK2	97
IKK-beta	100	MARK3	95
IKK-epsilon	100	MARK4	91
INSR	100	MAST1	75
INSRR	99	MEK1	89
IRAK1	81	MEK2	94
IRAK3	88	MEK3	84
IRAK4	73	MEK4	85
ITK	90	MEK5	87
JAK1(JH1domain-catalytic)	100	MEK6	99
JAK1(JH2domain-pseudokinase)	95	MELK	97
JAK2(JH1domain-catalytic)	60	MERTK	93
JAK3(JH1domain-catalytic)	86	MET	87
JNK1	100	MET(M1250T)	84
JNK2	88	MET(Y1235D)	92
JNK3	100	MINK	77
KIT	94	MKK7	97
KIT(A829P)	93	MKNK1	100
KIT(D816H)	95	MKNK2	87
KIT(D816V)	87	MLCK	98
KIT(L576P)	92	MLK1	90
KIT(V559D)	91	MLK2	100
KIT(V559D,T670I)	96	MLK3	100
KIT(V559D,V654A)	97	MRCKA	98
KIT-autoinhibited	84	MRCKB	99
LATS1	100	MST1	100
LATS2	100	MST1R	94
LCK	95	MST2	82
LIMK1	95	MST3	100
LIMK2	96	MST4	91
LKB1	100	MTOR	100
LOK	97	MUSK	93
LRRK2	86	MYLK	76
LRRK2(G2019S)	82	MYLK2	99
LTK	100	MYLK4	96
LYN	100	MYO3A	100
LZK	95	MYO3B	100
MAK	88	NDR1	82
MAP3K1	97	NDR2	100
MAP3K15	84	NEK1	100
MAP3K2	84	NEK10	93
MAP3K3	97	NEK11	84
MAP3K4	86	NEK2	96
MAP4K2	71	NEK3	86
MAP4K3	97	NEK4	90

NEK5	91	PIP5K1A	96
NEK6	100	PIP5K1C	96
NEK7	100	PIP5K2B	100
NEK9	100	PIP5K2C	85
NIK	91	PKAC-alpha	96
NIM1	90	PKAC-beta	100
NLK	93	PKMYT1	89
OSR1	97	PKN1	99
p38-alpha	96	PKN2	100
p38-beta	97	PKNB(M.tuberculosis)	78
p38-delta	88	PLK1	94
p38-gamma	69	PLK2	100
PAK1	100	PLK3	100
PAK2	94	PLK4	82
PAK3	100	PRKCD	82
PAK4	100	PRKCE	89
PAK6	96	PRKCH	91
PAK7	96	PRKCI	88
PCTK1	89	PRKCQ	81
PCTK2	96	PRKD1	82
PCTK3	100	PRKD2	97
PDGFRA	100	PRKD3	97
PDGFRB	77	PRKG1	100
PDPK1	100	PRKG2	94
PFCDPK1(P.falciparum)	78	PRKR	84
PFPK5(P.falciparum)	82	PRKX	82
PFTAIRE2	99	PRP4	89
PFTK1	100	PYK2	90
PHKG1	88	QSK	89
PHKG2	98	RAF1	43
PIK3C2B	80	RET	100
PIK3C2G	87	RET(M918T)	90
PIK3CA	85	RET(V804L)	94
PIK3CA(C420R)	91	RET(V804M)	97
PIK3CA(E542K)	98	RIOK1	100
PIK3CA(E545A)	95	RIOK2	96
PIK3CA(E545K)	92	RIOK3	94
PIK3CA(H1047L)	100	RIPK1	93
PIK3CA(H1047Y)	90	RIPK2	97
PIK3CA(I800L)	100	RIPK4	97
PIK3CA(M1043I)	72	RIPK5	97
PIK3CA(Q546K)	98	ROCK1	74
PIK3CB	93	ROCK2	76
PIK3CD	88	ROS1	99
PIK3CG	95	RPS6KA4(Kin.Dom.1-N-terminal)	99
PIK4CB	72	RPS6KA4(Kin.Dom.2-C-terminal)	98
PIKFYVE	97	RPS6KA5(Kin.Dom.1-N-terminal)	91
PIM1	96	RPS6KA5(Kin.Dom.2-C-terminal)	100
PIM2	100	RSK1(Kin.Dom.1-N-terminal)	100
PIM3	93	RSK1(Kin.Dom.2-C-terminal)	100

RSK2(Kin.Dom.1-N-terminal)	93	TIE2	96
RSK2(Kin.Dom.2-C-terminal)	88	TLK1	100
RSK3(Kin.Dom.1-N-terminal)	89	TLK2	100
RSK3(Kin.Dom.2-C-terminal)	94	TNIK	96
RSK4(Kin.Dom.1-N-terminal)	80	TNK1	72
RSK4(Kin.Dom.2-C-terminal)	99	TNK2	96
S6K1	86	TNNI3K	91
SBK1	94	TRKA	72
SGK	79	TRKB	83
SgK110	87	TRKC	98
SGK2	93	TRPM6	71
SGK3	100	TSSK1B	90
SIK	94	TSSK3	93
SIK2	100	TTK	94
SLK	94	TXK	91
SNARK	71	TYK2(JH1domain-catalytic)	52
SNRK	100	TYK2(JH2domain-pseudokinase)	83
SRC	95	TYRO3	72
SRMS	77	ULK1	98
SRPK1	100	ULK2	99
SRPK2	95	ULK3	86
SRPK3	94	VEGFR2	100
STK16	93	VPS34	91
STK33	91	VRK2	100
STK35	98	WEE1	100
STK36	100	WEE2	94
STK39	87	WNK1	96
SYK	94	WNK2	96
TAK1	91	WNK3	100
TAOK1	85	WNK4	100
TAOK2	71	YANK1	100
TAOK3	87	YANK2	88
TBK1	91	YANK3	94
TEC	92	YES	96
TESK1	85	YSK1	100
TGFBR1	59	YSK4	81
TGFBR2	100	ZAK	89
TIE1	100	ZAP70	67

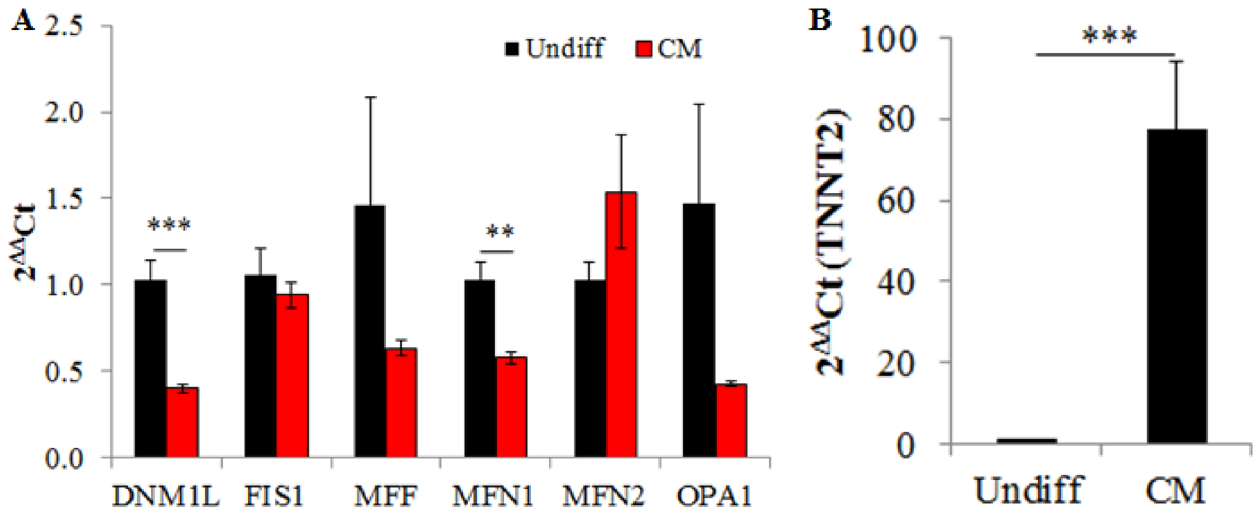

Supplementary Figure S1. Mitochondrial morphology of human iPSCs and derived cardiomyocytes in CERA007c6 cell line. (**A-B**) mRNA expression of mitochondrial fusion and fission genes (**A**), as well as cardiac-specific TNNT2 gene (**B**) in undifferentiated iPSCs (Undiff) and derived cardiomyocytes (CM) (n = 6 independent experiments). Data are shown as mean±SEM. ***P* < 0.01, ****P* < 0.001 by unpaired Student's *t*-test.

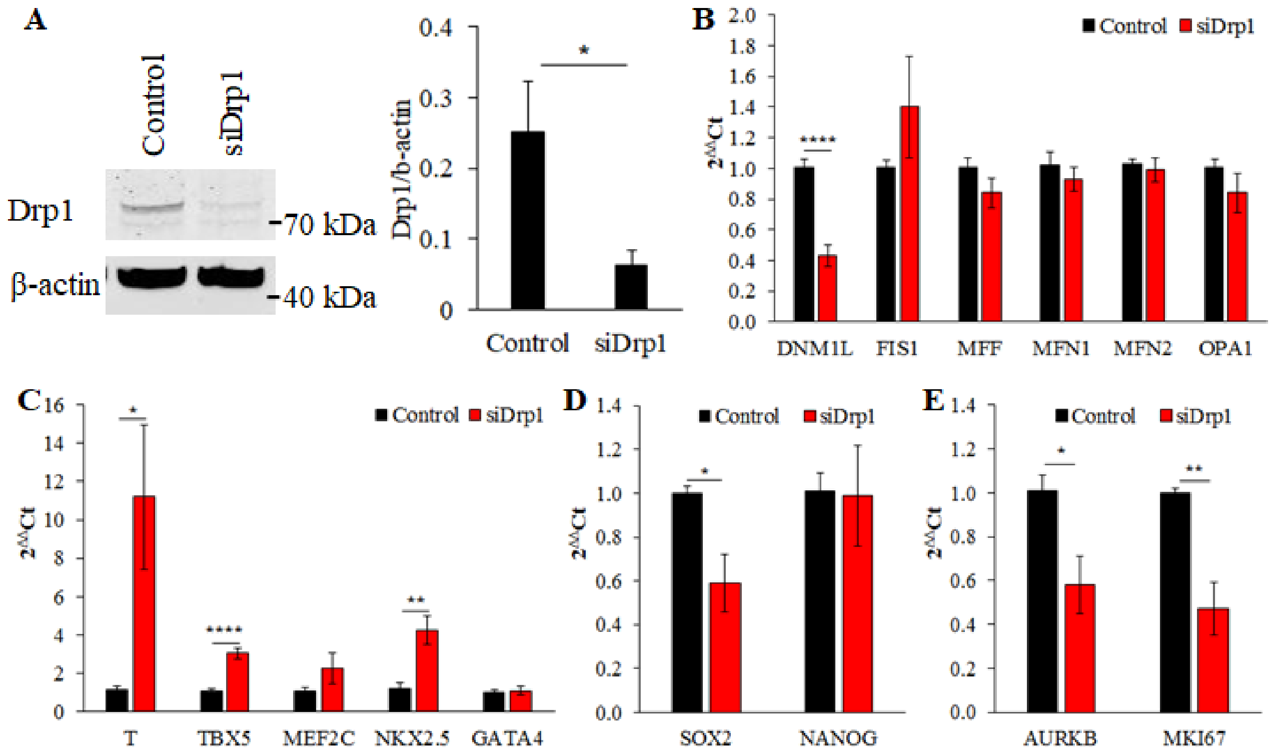

Supplementary Figure S2. Drp1 knock down in CERA007c6 iPSC line. (**A**) Western blotting analysis of Drp1 expression (n = 4 independent experiments). (**B-E**) mRNA expression of mitochondrial fusion and fission proteins (**B**), cardiac mesoderm transcription factors (**C**), pluripotency genes (**D**) and cell proliferation genes (**E**) in iPSCs treated with scrambled (control) or Drp1 siRNA (n = 6-7 independent experiments). Data are shown as mean±SEM. *$P < 0.05$, **$P < 0.01$, ****$P < 0.0001$ by unpaired Student's *t*-test.

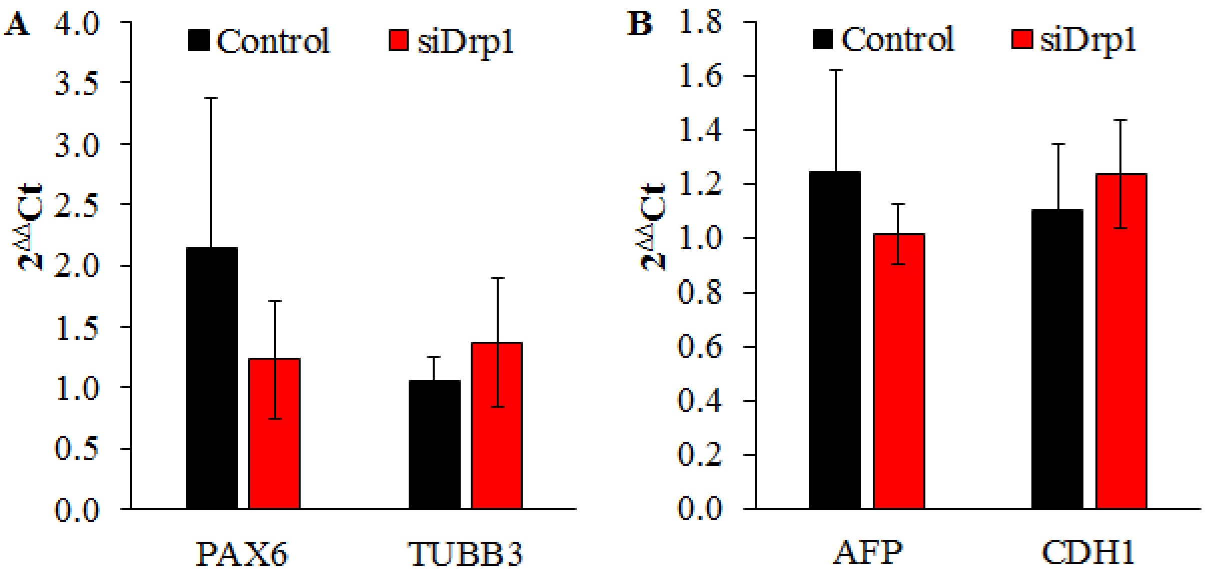

Supplementary Figure S3. Effect of Drp1 Knock down on ectodermal (*PAX6* and *TUBB3*) and endodermal (*AFP* and *CDH1*) gene expression in iPS-Foreskin-2 cell line. (n = 5 independent experiments). Data are shown as mean±SEM.

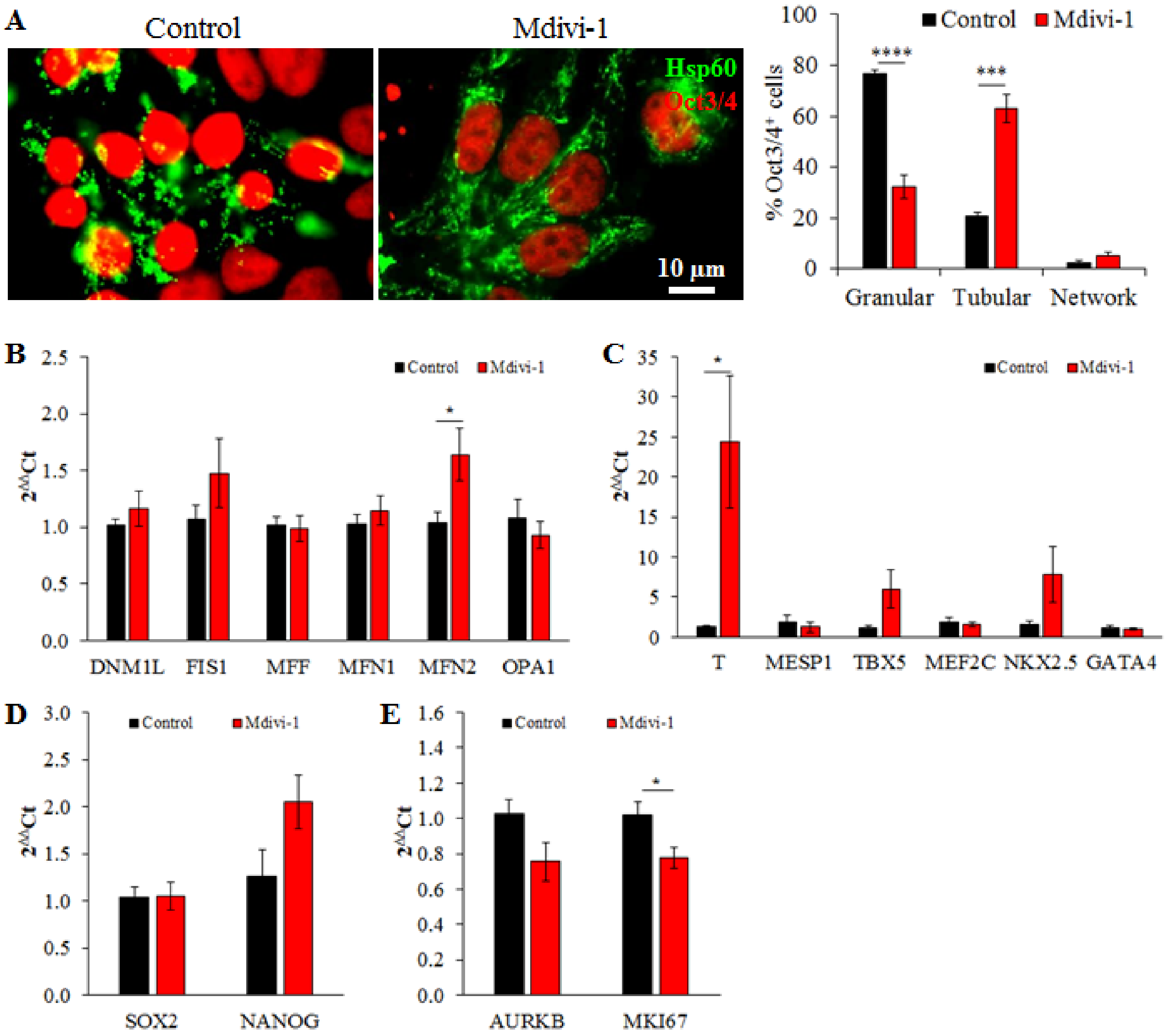

Supplementary Figure S4. Mdivi-1 induces cardiac mesoderm differentiation of human iPSCs. (**A**) Morphology of mitochondria and percentage of Oct3/4 positive iPS-Foreskin-2 cells with different mitochondrial morphologies (n = 4 independent experiment). (**B-C**) mRNA expression of mitochondrial fusion and fission protein (**B**), cardiac mesoderm transcription factors (**C**), pluripotency genes (**D**) and cell proliferation genes (**E**) in iPS-Foreskin-2 cells treated with DMSO (control) or 5 µM Mdivi-1 (n = 6-10 independent experiments). Data are shown as mean±SEM. **P* < 0.05, ****P* < 0.001, *****P* < 0.0001 by unpaired Student's *t*-test.

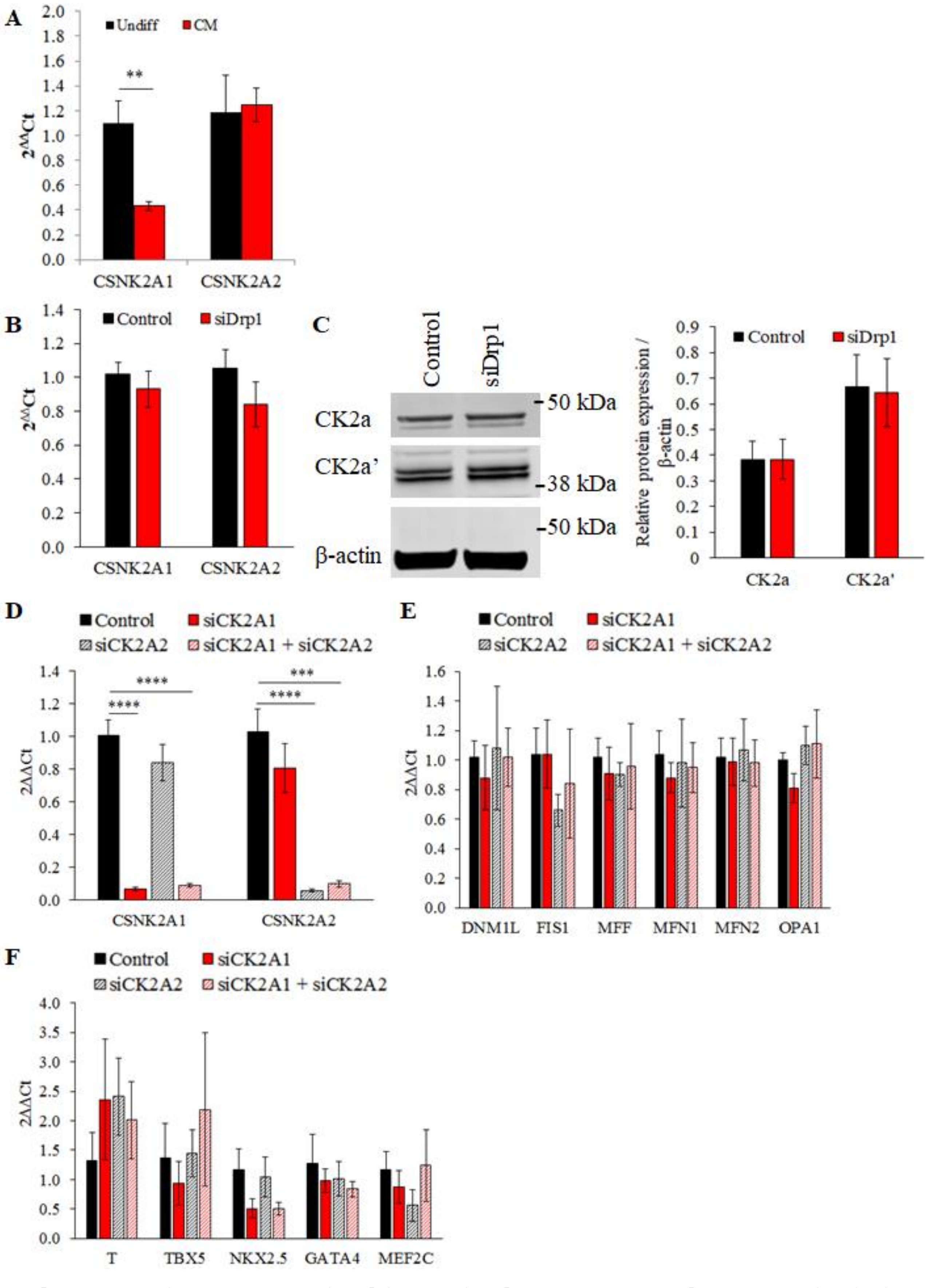

Supplementary Figure S5. Functional interaction between Drp1 and CK2 proteins in iPS-Foreskin-2 cell line. (**A**) mRNA expression of protein kinase CK2α (CSNK2A1) and CK2α' (CSNK2A2) in undifferentiated iPSCs (Undiff) and derived cardiomyocytes (CM) (n = 8 independent

experiments). (**B**) mRNA (n = 9 independent experiments) and (**C**) protein (n = 4 independent experiments) expressions of protein kinase CK2α and CK2α' in iPS(Foreskin)-2 cells treated with scrambled (control) or Drp1 siRNA. (**D-F**) mRNA expression of protein kinase CK2α and CK2α' (**D**), mitochondrial fusion and fission genes (**E**), and cardiac mesoderm transcription factors (**F**) in iPSCs treated with scrambled (control), CK2α (siCK2A1), CK2α' (siCK2A2) or both CK2α and CK2α' siRNA (n = 4 independent experiments). Data are shown as mean±SEM. $^{**}P < 0.01$, $^{***}P < 0.001$, $^{****}P < 0.0001$ by one-way ANOVA with the Bonferroni post hoc test.

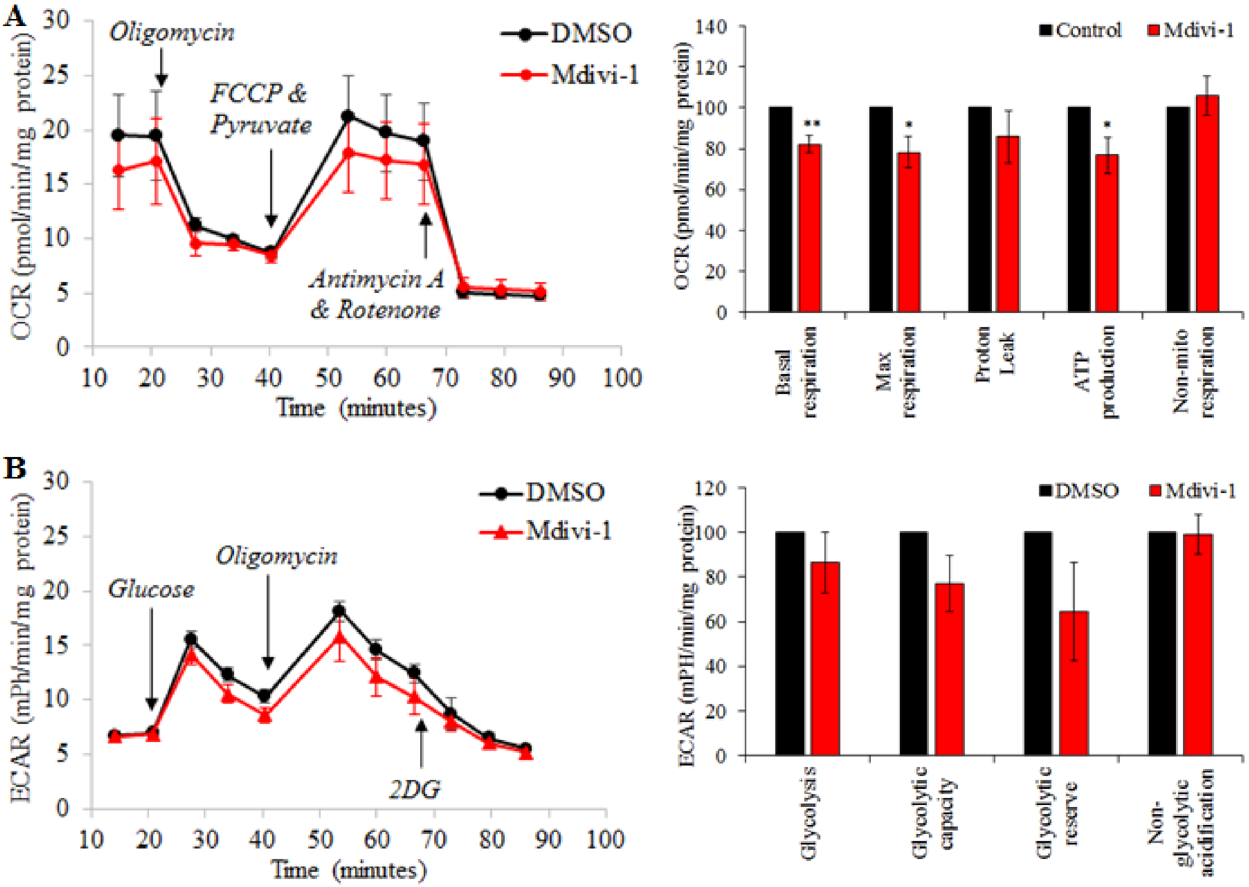

Supplementary Figure S6. Effect of Mdivi-1 treatment on mitochondrial respiration and glycolysis. (**A**) Oxygen consumption rate (OCR) and (B) extracellular acidification rate (ECAR) of iPS-Foreskin-2 cells treated with DMSO (control) or 5 μM Mdivi-1 (n = 4 independent experiments). Data are shown as mean±SEM. *P < 0.05, **P < 0.01 by unpaired Student's t-test.

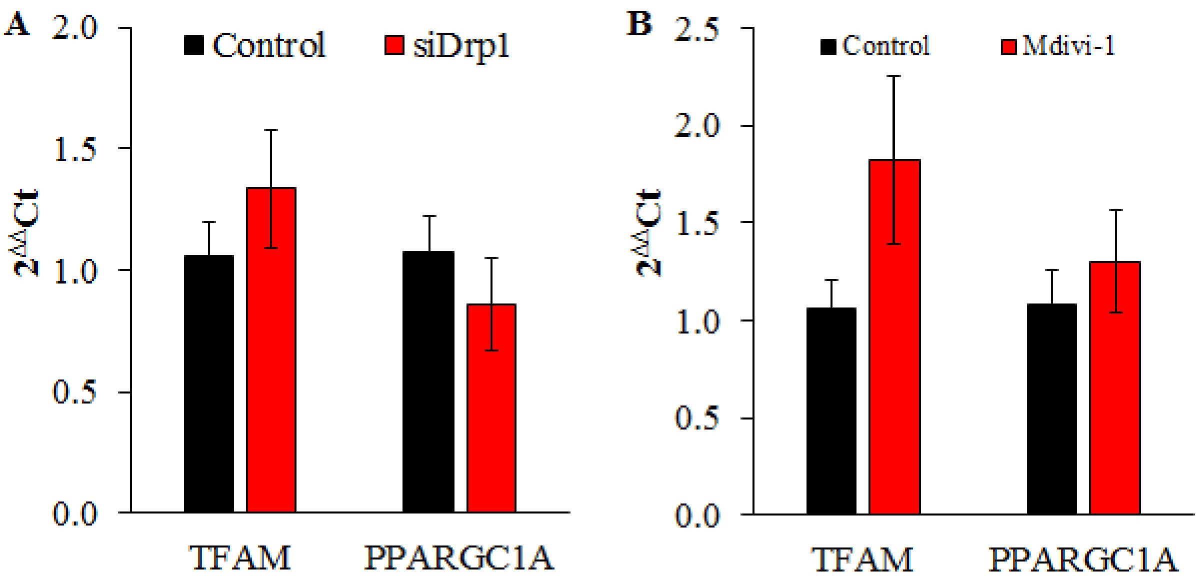

Supplementary Figure S7. Effect of Drp1 Knock down (A) and Mdivi-1 treatment (B) on gene expression of *TFAM* and *PPARGC1A* in iPS-Foreskin-2 cell line. (n = 7 independent experiments). Data are shown as mean±SEM.
